# Determinants of Parental Attitudes towards Children’s Vaccination in Lithuania: An Online Survey

**DOI:** 10.15388/Amed.2024.31.1.7

**Published:** 2024-02-27

**Authors:** Kamilė Čeponytė, Eglė Narkevičiūtė, Aušra Beržanskytė, Sigita Burokienė

**Affiliations:** 1 Faculty of Medicine, Vilnius University, Vilnius, Lithuania; 2 Department of Public Health, Institute of Health Sciences, Faculty of Medicine, Vilnius University, Lithuania; 3 Clinic of Children’s Diseases, Institute of Clinical Medicine, Faculty of Medicine, Vilnius University, Lithuania

**Keywords:** parental attitudes, vaccination, infectious diseases, socioeconomic variables, demographic factors, tėvų požiūris, vakcinacija, infekcinės ligos, socioekonominiai kintamieji, demografiniai veiksniai

## Abstract

**Background:**

Data from recent years show that the COVID-19 pandemic has significantly reduced the scope of child immunisation worldwide. If the numbers continue to fall, we may have a vaccination crisis. In order to understand the local factors of this phenomenon, we studied parents‘ viewpoint towards childhood vaccination in Lithuania. Therefore, the aim of our study was to determine the impact of parental attitudes on children’s vaccination.

**Materials and Methods:**

A web-based cross-sectional study was made in Lithuania from October 20th to November 3rd, 2020, using Google Forms. An anonymous questionnaire included both quantitative and qualitative questions. The questionnaire was distributed to the general population in Lithuania via social media and a snowball sampling. Only parents who have at least one child under 18 years old were involved in the study. We analysed the parents’ attitudes towards vaccination by their socioeconomic and demographic determinants using MS Excel and OpenEpi tools. The respondents were considered vaccine supporters if they vaccinated all their children with state-reimbursed vaccines or only vaccinated some of their children, and vaccine sceptics if they did not vaccinate their children with state-reimbursed vaccines.

**Results:**

The population of our research consisted of 775 parents. There were more males against vaccination than females, respectively, 59.6% and 33.2%. 65.0% of all respondents stated that they vaccinated their children with the full course of vaccines, while the remaining 35.0% did not vaccinate. University-level education dominated in both pro-vaccine and anti-vaccine groups. We found that 16.9% of parents who were unvaccinated as children themselves tend not to vaccinate their children. However, in the pro-vaxx group, only 0.7% of parents were unvaccinated during childhood. 50.8% of vaccine sceptics were not fully informed by healthcare professionals about the benefits and risks of vaccination, while only 31.6% of vaccine supporters were not informed. Social environment did not have an impact on the decision whether to vaccinate or not to vaccinate a child among both vaccine advocates and vaccine deniers (accordingly, 51.2% and 42.9%). 29.3% of respondents stated that the cost of paid vaccinations was too high. Open-text survey responses gave us more in-depth insight about the parental decision-making process. Protection of children and society from infectious diseases (31.7%) was mentioned as the main reason for vaccination. Whereas vaccine-hesitant parents were mostly concerned about already-occurring side effects or their risk (43.4%).

**Conclusions:**

Our findings confirmed that vaccine hesitancy was associated with not being vaccinated in childhood themselves, lack of information from medical practitioners and male gender. The price of vaccines also had an impact on immunisation rates – more than one-third of the vaccine supporting parents indicated that the cost of paid vaccinations was too high. The main incentive for vaccine compliance was parents’ desire to protect their children and society from infectious diseases. Whereas vaccine-hesitant parents were mostly concerned about already occurring side effects or their risk. There was no consensus, whether vaccination of children should be mandatory in Lithuania, as the answers to the question were almost evenly distributed. The formation of parents‘ attitudes towards children‘s vaccination is a complex process that is determined by parents‘ attitudes towards the health care system, lack of trust in doctors, and gaps in communication about the benefits and risks of vaccination. All of this information should be taken into account in health policy-making.

## Abstract (it)

**Įvadas:**

Pastarųjų metų duomenys rodo, kad COVID-19 pandemija labai sumažino vaikų imunizacijos mastą visame pasaulyje. Jei skaičiai ir toliau mažės, gali kilti vakcinacijos krizė. Siekdami suprasti lokalias šio reiškinio priežastis, tyrėme tėvų požiūrį į skiepijimą Lietuvoje. Todėl šiuo tyrimu buvo siekiama nustatyti tėvų požiūrio įtaką vaikų skiepijimui.

**Metodai:**

Lietuvos populiacijoje buvo atliktas skerspjūvio tyrimas nuo 2020 m. spalio 20 d. iki lapkričio 3 d., internete, naudojant ,,Google“ formas. Anoniminėje anketoje buvo pateikti tiek kiekybiniai, tiek kokybiniai klausimai. Anketa buvo išplatinta per socialinius tinklus bei naudojant „sniego gniūžtės“ atranką. Tyrime galėjo dalyvauti tik tėvai, turintys bent vieną vaiką iki 18 metų amžiaus. Gautus duomenis apie tėvų požiūrį į vaikų skiepijimą pagal socialinius, ekonominius ir demografinius veiksnius išanalizavome naudodami MS Excel ir OpenEpi įrankius. Respondentai buvo laikomi skiepų šalininkais, jei jie skiepijo savo vaikus valstybės kompensuojamomis vakcinomis arba skiepijo tik kai kuriuos vaikus. Tuo tarpu, vakcinacijos oponentais – jei neskiepijo savo vaikų valstybės kompensuojamomis vakcinomis.

**Rezultatai:**

Mūsų tyrime dalyvavo 775 tėvai. Vakcinacijos oponentų vyrų buvo daugiau nei moterų, atitinkamai 59.6% ir 33.2%. 65.0% visų apklaustųjų nurodė, kad savo vaikus skiepija visu skiepų kursu, o likę 35.0% – neskiepijo. Universitetinis išsilavinimas dominavo tiek vakcinuojančių, tiek nevakcinuojančiųjų grupėse. Nustatėme, kad 16.9% tėvų, kurie patys vaikystėje buvo neskiepyti, yra linkę neskiepyti savo vaikų. Tačiau vakcinuojančiųjų grupėje tik 0.7% tėvų buvo neskiepyti vaikystėje. 50.8% vakcinacijos skeptikų teigė, jog sveikatos priežiūros specialistai neinformavo jų apie skiepijimo naudą ir riziką, tuo tarpu tik 31.6% vakcinacijai pritariančiųjų nebuvo informuoti. Socialinė aplinka neturėjo įtakos apsisprendimui, ar skiepyti vaiką tiek tarp skiepų šalininkų, tiek skiepų oponentų (atitinkamai: 51.2% ir 42.9%). 29.3% respondentų teigė, kad mokamų skiepų kaina yra per didelė. Atviro klausimo atsakymai suteikė mums išsamesnės informacijos apie tėvų sprendimų priėmimo procesą. Pagrindinis skiepijimo motyvas buvo vaikų ir visuomenės apsauga nuo infekcinių ligų (31.7%). Tuo tarpu skiepyti nesiryžę tėvai nerimavo dėl jau pasireiškusių šalutinių poveikių ar jų rizikos (43.4%).

**Išvados:**

Mūsų išvados patvirtino, kad nenoras skiepyti savo vaikų buvo susijęs su pačių tėvų vakcinacijos statusu vaikystėje, informacijos iš gydytojų stoka ir vyriška lytimi. Skiepijimo apimtims įtakos turėjo ir skiepų kaina – daugiau nei trečdalis skiepijimui pritariančių tėvų nurodė, kad mokamų skiepų kaina yra per didelė. Pagrindinė paskata skiepyti buvo tėvų noras apsaugoti savo vaikus ir visuomenę nuo infekcinių ligų. Tuo tarpu vakcinacijos oponentai dažniausiai nerimavo dėl jau pasireiškusių šalutinių poveikių ar jų rizikos. Vieningos nuomonės, ar Lietuvoje privaloma skiepyti vaikus, nebuvo, nes atsakymai į klausimą pasiskirstė beveik po lygiai. Tėvų požiūrio į vaikų skiepijimą formavimasis yra sudėtingas procesas, kurį lemia tėvų požiūris į sveikatos priežiūros sistemą, nepasitikėjimas gydytojais bei komunikacijos apie skiepijimo naudą ir riziką spragos. Į visus šiuos aspektus reikėtų atsižvelgti formuojant sveikatos politiką.

## Introduction

The 21st century brings us new challenges in the field of infectious disease control. The mere fact that vaccine hesitancy was recognised by the WHO as one of the ten threats to global health in 2019 underlines the magnitude of the problem and encourages immediate action (1). At the end of 2021, the WHO called for revitalising efforts to tackle communicable diseases in the world because routine immunisation against other infectious diseases was unavailable to many people during the COVID-19 pandemic (2). According to the preventive vaccination report of the National Public Health Center under the Ministry of Health, in Lithuania, as in the whole world, the scope of childhood vaccinations dropped (3). If the issue of declining vaccination coverage is not resolved in time, we can reverse the progress achieved over centuries in the elimination of transmissible diseases or even have new epidemic outbreaks that could have been completely prevented by vaccines. WHO presents complacency, inconvenience in accessing vaccines, and lack of confidence as the three most important reasons for vaccine hesitancy (1). The aim of our study was to determine the influence of parental attitudes on children’s vaccination.

## Materials and methods

### Study design and participants

Data for our web-based cross-sectional study were collected in Lithuania from October 20th to November 3rd, 2020, during the period between two national lockdowns. The questionnaire was distributed to the general population in Lithuania via social media platforms. Also, personal contacts via e-mails and snowball sampling were used, i.e., participants were asked to assist researchers in disseminating the questionnaire to other potential respondents. Only parents with at least one child under the age of 18 were involved in the study. The main information, the purpose, and the design of the study were presented at the beginning of the online questionnaire, and respondents had to give consent in order to participate anonymously in the research. Taking into consideration the margin error of ± 5% and the confidence level of 95%, a sample size of 384 was calculated as sufficient. Therefore, our sample size of 775 respondents was acceptable.

### Questionnaire

We constructed the original questionnaire by taking into account some questions from Facciolà A et al. (4) and Coniglio MA et al. (5) studies that have been done on this topic previously. The questionnaire was shared using Google Forms, which allowed for convenient distribution and guaranteed anonymity. We divided the questionnaire into four sections: socioeconomic and demographic data section (1–7 questions), child’s immunisation status (8–10 questions), reasons determining vaccination choice (11; 13–15 questions), and lastly, parental immunisation status and opinion on mandatory vaccination (12 and 16 questions). For a deeper insight into children’s vaccination, an open question was designed in the third section: “What reasons led you to make the choice whether or not to vaccinate your child(ren)?”

### Our questionnaire consisted of the following questions:

1. What is your gender?
2. What is your age?
3. What is your education?
4. What is your monthly income (EUR)?
5. What is your living area?
6. How many children do you have?
7. To what educational institution does your child go to?
8. Do you vaccinate your child with state-reimbursed vaccinations?
9. Have you vaccinated your child(ren) for the full course of vaccinations?
10. Do you vaccinate your child(ren) with additional preventive vaccinations that are not financed by the state?
11. What reasons led you to make the choice whether or not to vaccinate your child(ren)? (Text answer)
12. Were you vaccinated as a child with state-reimbursed and additional preventive vaccines that were not compensated by state funds?
13. Have you been fully informed by the medical staff about the benefits and risks of vaccinating children?
14. Was your decision to vaccinate or not vaccinate your child(ren) influenced by the environment (e.g., the opinions of public figures, social media, relatives, neighbours, friends, or doctors)?
15. Does the price of paid vaccinations affect your decision to vaccinate your child(ren)?
16. In your opinion, should prophylactic vaccination of children be mandatory in Lithuania?

A pilot study of 20 participants was done before the main study in order to test the feasibility and quality of the questionnaire. Corrections were made according to the participants’ feedback.

### Data analysis

The main variable measured was whether parents vaccinate their children with state-reimbursed vaccinations. The respondents were considered vaccine supporters if they vaccinated all their children with state-reimbursed vaccines or only vaccinated some of their children, and vaccine sceptics if they did not vaccinate their children with state-reimbursed vaccines. We performed descriptive analysis by collecting variables such as socioeconomic and demographic factors such as gender, age, education level, income, residence, number of children in the family, child’s educational institution, child’s and parent’s immunisation status, attitude towards vaccination, information given to parents by medical specialists, environmental influence on the decision to vaccinate, and attitude towards paid vaccinations. We analysed the parents’ attitudes towards vaccination based on their socioeconomic and demographic determinants using MS Excel and OpenEpi tools. The relative frequencies (%) with 95% confidence intervals (CI) were calculated for the prevalence of chosen variables in vaccine supporters and vaccine sceptics groups. The Chi-squared test with a significance threshold of p=0.05 was applied to assess statistical diversions between those groups. The open question gave us more in-depth insights about parents’ approaches. We scrutinised it by manually performing text analysis. Our text analysis was based on three main steps: data collection, structuring, and evaluation of the results. Furthermore, we combined similar answers into categories and calculated their frequencies.

## Results

Data that reflects our respondents’ socioeconomic and demographic characteristics are presented in Table 1.

**Table 1:** Socio-economic and demographic characteristics of respondents

Characteristics	N	%
**Sex**		
Male	52	6.7
Female	723	93.3
**Age groups**		
<18	4	0.5
18–28	174	22.5
29–39	470	60.6
40–50	109	14.1
51–61	15	1.9
≥62	3	0.4
**Level of education**		
Incomplete secondary education	4	0.5
Secondary education	40	5.2
Vocational education	39	5.0
College	129	16.6
University	555	71.6
Other	8	1.0
**Monthly income (EUR)**		
<500	66	8.5
500–1000	275	35.5
1001–1500	227	29.3
1501–2000	100	12.9
≥2001	107	13.8
**Residence**		
City (>3000 residents)	626	80.8
Town (500–3000 residents)	87	11.2
Village (<500 residents)	58	7.5
Other	4	0.5
**Number of children in family**		
1	385	49.7
2	303	39.1
≥3	87	11.2
**Child education institution**		
Public kindergarten	289	37.4
Private kindergarten	117	15.1
Public school	214	27.7
Private school	30	3.9
Education of preschool age child at home	145	18.8
Education of a school-age child at home	7	0.9
Other	121	15.7

The vast majority of responding parents consisted of females, 723 (93.3%), while there were only 52 (6.7%) males. Most of the participants in the survey were middle-aged parents, ranging in age from 29 to 39 years old. Our respondents tend to have a higher university education level (71.6%), whereas only 4 parents claimed they haven’t completed secondary education. The typical average monthly income of one of the parents varies from 500 to 1000 euros. The absolute majority of respondents claimed to be living in the city (80.8%). A high proportion of survey participants reported that they have 1 or 2 children in their family (accordingly, 49.7% and 39.1%). 18.8% of preschool children are educated at home by their parents, but there is a clear trend that the vast majority of parents choose to send their children to a public kindergarten (37.4%) or school-age children to a public school (27.7%).

Even 65.0% (95% CI: 61.6–68.3) of parents vaccinated their children with the full course of vaccines, while the remaining 35.0% (95% CI: 31.7–38.4), p<0.001, were hesitant (i.e., they did not vaccinate or did not fully vaccinate their children). Data that reflects vaccine supporters’ and sceptics’ traits is presented in Table 2.

**Table 2:** Portrait of vaccine supporters and sceptics

	Vaccine supporters, N/(%) N=586		Vaccine sceptics, N/(%) N=189		p value
Characteristics	N	% (95% CI)	N	% (95% CI)	
**The child received a complete vaccination course**					
Yes	499	85.2 (82.1–87.9)	5	2.7 (1.0–5.8)	p<0.001
No	87	14.9 (12.1–17.9)	184	97.4 (94.2–99.0)	
**Environmental influence on the choice of whether to vaccinate a child or not**					
No, I do not care about other people’s opinions	300	51.2 (47.2–55.2)	81	42.9 (35.9–50.0)	p=0.064
Partly, it had an impact	172	29.4 (25.8–33.1)	72	38.1 (31.4–45.2)	
Yes, it had an impact	114	19.5 (16.4–22.8)	36	19.1 (13.9–25.1)	
**Immunization status of the respondents**					
Vaccinated with state-reimbursed vaccinations	455	77.7 (74.1–80.9)	142	75.1 (68.6–80.9)	p<0.001
Vaccinated with both state-reimbursed and nonstate-reimbursed vaccinations	112	19.1 (16.1–22.5)	5	2.7 (1.0–5.8)	
Vaccinated with only nonstate-reimbursed vaccinations	15	2.6 (1.5–4.1)	10	5.3 (2.7–9.2)	
Not vaccinated	4	0.7 (0.2–1.6)	32	16.9 (12.1–22.8)	
**Information provided by medical staff to parents about the benefits and risks of vaccinating children**					
Yes, I was fully informed	161	27.5 (24.0–31.2)	34	18.0 (13.0–24.0)	p<0.001
No, I was not fully informed	185	31.6 (27.9–35.4)	96	50.8 (43.7–57.9)	
I was partly informed	240	41.0 (37.0–45.0)	59	31.2 (24.9–38.1)	
**Child’s vaccination with nonstate-reimbursed vaccinations**					
Yes	354	60.4 (56.4–64.3)	3	1.6 (0.4–4.3)	p<0.001
No	232	39.6 (35.7–43.6)	186	98.4 (95.7–99.6)	
**The choice of whether to vaccinate is influenced by the cost of vaccinations**					
Yes, it is too expensive, I do not vaccinate	50	8.5 (6.5–11.01)	0	0 (0.0–1.6)	p<0.001
Yes, it is too expensive, but I still choose to vaccinate	176	30.0 (26.4–33.8)	1	0.5 (0.0–2.6)	
No, I do vaccinate, regardless of the price	244	41.6 (37.7–45.7)	4	2.1 (0.7–5.0)	
No, I do not vaccinate due to my opinion	116	19.8 (16.7–23.2)	184	97.4 (94.2–99.0)	
**The vaccination of children should be mandatory in Lithuania**					
Yes	373	63.7 (59.7–67.5)	2	1.1 (0.2–3.5)	p<0.001
No	149	25.4 (22.0–29.1)	186	98.4 (95.7–99.6)	
I do not know	64	10.9 (8.6–13.6)	1	0.5 (0.0–2.6)	

59.6% of males were in the anti-vaccine group, while only 33.2% of females were against vaccination. Our results suggest that the environment (e.g., the opinions of public figures, social media, relatives, neighbours, friends, and doctors) did not have an influence on the decision whether to vaccinate or not to vaccinate a child for both pro-vaxxers (51.2%; 95% CI: 47.2–55.2) and anti-vaxxers (42.9%; 95% CI: 35.9–50.0), p=0.064. There were more respondents who had not been vaccinated in their childhood in the vaccine sceptics group in comparison to the vaccine supporters’ group, respectively, 16.9% (95% CI: 12.1–22.8) and 0.7% (CI: 0.2–1.6), p<0.001. 38.5% of the vaccine-supporting parents indicated that the cost of paid vaccinations was too high. Despite that, even 60.4% (95% CI: 56.4–64.3), p<0.001, of the respondents in the pro-vaccine group chose to vaccinate their children with nonstate-reimbursed vaccinations. We therefore investigated the potential association of information given to parents by healthcare professionals with their children vaccination status: 50.8% (95% CI: 43.7–57.9) of those vaccine sceptics were not fully informed by medical practitioners about the benefits and risks of vaccination, while only 31.6% (95% CI: 27.9–35.4), p<0.001, of vaccine supporters were not informed. Taking into account the replies of all respondents to the question of whether vaccination of children should be mandatory in Lithuania, positive and negative answers were almost evenly distributed: 48.4% for and 43.2% against. Meanwhile, a small number (8.4%) of parents did not have a strong opinion on this issue. Nevertheless, in the vaccine supporters’ group, 63.7%, and in the vaccine sceptics group, only 1.0% are in favour of compulsory vaccination of children in Lithuania.

**Fig. 1:**
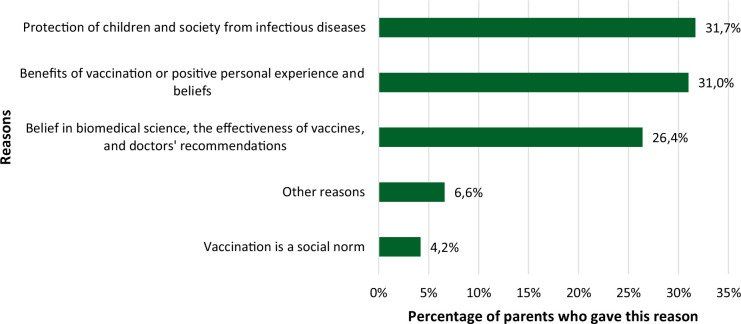
Reasons for parental vaccine acceptance

Based on the answers to the question “What reasons led you to make the choice whether or not to vaccinate your child(ren)?”, we divided participants’ responses about parental vaccine acceptance or hesitancy into separate groups by reasons. In total, there are five groups of reasons for vaccination and ten groups against it. The following reasons were stated for parental vaccine acceptance (Fig. 1).

As demonstrated, the most repeated answers were (Fig. 1): protection of children and society from infectious diseases – 31.7% (e.g., *“I was interested in vaccinations, the prevalence of diseases, the consequences of getting sick. My logic is simple, if I can vaccinate against a deadly disease or potentially cause serious complications – I vaccinate. In order to protect both my children and the population and those who cannot be vaccinated.”)*; benefits of vaccination or positive personal experience and beliefs – 31.0% (e.g., *“I vaccinate the child with time-proven vaccines that have been around for years and their benefits have been proven.”)* and belief in biomedical science, the effectiveness of vaccines, and doctors’ recommendations – 26.4% (e.g., *“If vaccinations are mandatory, then those diseases are the most dangerous for a child’s life”)*.

**Fig. 2:**
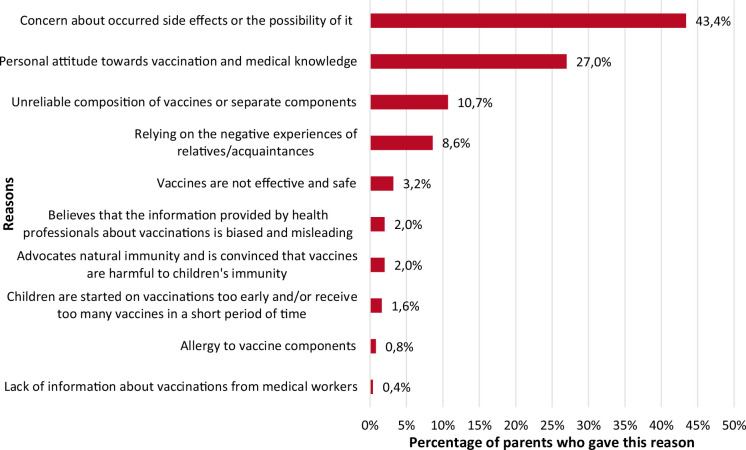
Reasons for parental vaccine nonacceptance

The following reasons were given for parental vaccine hesitancy by survey participants (Fig. 2).

The most frequent reasons that were chosen by vaccine-hesitant parents are as follows (Fig. 2): concern about occurred side effects or the possibility of them – 43.4% (e.g., *“The vaccinated child was sick non-stop. When I stopped vaccinating, he stopped getting sick. I did not vaccinate my second child, he is not sick at all. It is clear that vaccinations damage the immune system and make it unable to fight back. I know many such cases in my environment.”*; personal attitude towards vaccination and medical knowledge – 27.0% (e.g., *“Even before the child was born, I became interested and decided that the consequences of vaccines scare me more than the disease itself.”; “Facts given by many doctors/specialists, parents’ stories, untouchability of vaccine companies for defective product, vaccine ingredients such as aborted babies, mercury, polysorbate-80, MSG, egg embryo, etc...”)*; unreliable composition of vaccines or separate components – 10.7% (e.g., *“My interest in the composition of vaccinations, their necessity/unnecessity. The realisation that natural immunity is the most important thing for a small child! Realising why there are so many allergic kids around with horrible rashes, etc. I’m not against vaccines, but the amounts for a small baby are ABNORMAL!”)*.

## Discussion

### Statement of major findings

A web-based cross-sectional study revealed multiple main findings. To begin with, contrary to our primary hypothesis, environmental influence did not play a large role in the parental decision-making process of whether to vaccinate a child. Our data revealed that the main factors of vaccine compliance remain as follows: protection of children and society from infectious diseases, benefits of vaccination or positive personal experience and beliefs, trust in biomedical science, the effectiveness of vaccines, and doctors’ recommendations. Moreover, a clear tendency was noticed: parents who were unvaccinated themselves tend not to vaccinate their children either or are more likely to support the anti-vaxxer movement. The results of our research suggest that healthcare professionals play a huge role in the decision-making process, especially for vaccine sceptics. Online survey findings imply that parents did not receive enough information about vaccination risks and benefits. The observation is notable as it indicates that there is a window for opportunity in clinical communication between medical staff and patients. Despite the fact that discussion about compulsory vaccination has been a burning issue for a while, still no unanimity was reached owing to differing opinions that were divided nearly equally. During our survey, the data obtained from a close-ended question (“Have you been fully informed by the medical staff about the benefits and risks of vaccinating children?”) was surprisingly contradictory to the open-text question “What reasons led you to the choice whether or not to vaccinate your child(ren)?”. That is, the first mentioned question showed an association between the lack of information about vaccinations received from medical workers and vaccine hesitancy. Meanwhile, in the open question, lack of information was the least frequently mentioned reason for choosing not to vaccinate. There may be several reasons for this discrepancy, such as social desirability bias or the fact that the other motives appear more important to the respondents, e.g., the unreliable composition of vaccines or side effects. This finding implies that further research is needed.

### Comparison with relevant literature

In Lithuania, parental attitudes towards children’s vaccination have not been widely studied. For example, some of our findings are quite similar to a novel study by Čiutienė, 2022 (6). To begin with, her study indicated that sociodemographic factors such as educational status and monthly income had no statistical significance on childhood vaccination frequency (6). Our findings about male gender association with vaccine hesitancy correspond to Čiutienė study results, which state that there is statistical significance between gender and attitude towards vaccination (6). The general outcome of our study is also in line with the published results of Čiutienė: the main reason for vaccine noncompliance is a concern about side effects (6). This leads us to the point that vaccine safety in general remains a major parents’ concern. In addition, a large number of respondents claimed that their medical knowledge or a personal attitude was the reason why they refused to vaccinate their children. The same tendency was seen in our and the mentioned study: social environment does not have an impact on parents’ choice towards children’s immunisation (6). Given this finding, it can be said that the study results contradict our primary hypothesis about the immense environmental role in parental decision about whether to vaccinate their child. Nonetheless, an important factor that affects the scope of vaccination in Lithuania is the price of vaccines. Our study data is consistent with Žagminas et al. about the influence of price on vaccination rates: approximately only half of the parents who took part in the survey are able and willing to pay for nonreimbursed vaccines themselves (7). Unfortunately, our results demonstrate that for almost a third of parents, vaccine prices are too high. Thus, it is worth discussing whether the state’s choice to reimburse more vaccinations for children would increase vaccine coverage in the near future. Previous works by Ebi et al., My et al., and Žagminas et al. have shown that the main sources of information on childhood immunisation are healthcare institutions and primary care physicians (7; 8; 9). Nevertheless, quite a few respondents indicated a lack of knowledge about childhood immunisation. In addition to this, in our study, an alarming trend was noticed: even 36.2% of parents stated that they were not comprehensively informed by medical practitioners about vaccination benefits and risks, while 38.7% were partly informed, and just 25.1% confirmed that they were fully informed.

### Implications

The optimal 90–95% childhood vaccination coverage is recommended by the National Public Health Center under the Ministry of Health (10).

According to their 2019–2021 data, 90% vaccination coverage was achieved only against tuberculosis and hepatitis B, whereas other recommended childhood vaccines did not exceed this line (10). Our study results also show a similar trend of declining vaccination rates, as only 65.0% of parents made a decision to vaccinate their children with the full course of vaccines. We distinguish that vaccine scepticism might be associated with fear of vaccine side effects or untrustworthy composition, personal viewpoint on immunisation and insufficient information provided by medical practitioners. All these factors can lead to vaccine delaying, vaccine hesitancy, or even noncompliance with the recommended schedule, and action must be taken to reduce their negative impact on the scope of children’s vaccinations. This brings us to recommendations on boosting childhood immunisation rates and minimising the risk of contracting vaccine-preventable diseases. Our perspective matches the outlook of other authors, who believe that there is no single effective child vaccination policy suitable for all countries. Considering that, it is extremely important to take into account the socioeconomic situation of the country, the prevailing cultural attitudes, the current level of immunisation of children and outbreaks of infectious diseases, and the availability of vaccination services. We propose that Lithuania is still not ready for the implication of mandatory vaccination, as a lot of residents are still in doubt. In order to predict the possible impact of a mandatory vaccination policy, it is most appropriate to rely on the experience of similar countries where this policy has already been introduced. Focusing on alternative instruments, increasing trust between parental figures and pediatricians should be established through clear communication because medical professionals still remain the most important source of information (9; 11). In order to achieve that, clinical communication skills training for healthcare workers would be beneficial. To help clarify parental concerns and close information gaps, different evidence-based tools, such as fact sheets about vaccine safety and possible side effects, organising educational lectures on vaccination benefits and risks, or creating vaccine schedule tracking mobile apps would be beneficial (9). Importantly, strategies ought to be of a guiding and nondirective style to let the parents come to their conclusions on their own terms (9). Therefore, while adopting a national vaccination policy, the goal should be an optimal balance between parental autonomy in the decision-making process and public health authorities.

### Unanswered questions and future research

There are a number of important questions for further research and discussion. We strongly suggest designing a future study based on our current work and experience in a broader context, e.g., to conduct research in other Baltic countries on parental attitudes towards children’s vaccination and compare the collected data with those in Lithuania. It will also be beneficial for researchers to investigate how the COVID-19 pandemic affected parents’ compliance with the national immunisation program. What are the main triggers that turn vaccine-hesitant or undecided parents into a whole separate anti-vaccination movement? How can medical professionals reach this growing group of people and gain their trust to provide them with science-based information? How to measure parents’ satisfaction level with primary healthcare providers?

### Strengths of this study

There is a lack of data and scientific work on vaccine hesitancy, especially in the Baltic region during the COVID-19 pandemic. Moreover, the analysis of parental vaccine noncompliance allowed us to look at the problem as an interdisciplinary research field and identify how it is affected by various factors, such as socioeconomic or demographic. Besides, the study sample size was large enough (775 respondents) to provide sufficient levels of certitude.

### Limitations of this study

The findings of this study have to be seen in light of some limitations. First of all, due to the unexpected introduction of COVID-19 restrictions in Lithuania, questionnaires for parents were distributed only on social networks and via email (i.e., personal contacts and snowball sampling). As a result, potential selection bias might exist. Secondly, it would be beneficial to inquire with parents about their main information sources on childhood immunisation. Thirdly, few men participated in our survey, so the sample size may not reflect the correct proportion of the population in Lithuania by sex ratio. Though in other similar surveys of parents, the majority of participants were also women. Thus, we can assume that the opinion of one family representative (in our case, usually the mother) most often reflects the attitude of the whole family towards the vaccination of children and other questions.

## Conclusions

The vaccine hesitancy among parents in Lithuania was associated with not being vaccinated in childhood themselves, a lack of information from medical practitioners and male gender. The price of vaccines also had an impact on immunisation rates – more than one-third of the vaccine supporting parents indicated that the cost of paid vaccinations was too high. However, parental educational status or environmental influence did not have a statistical significance. The main incentive for compliance with vaccination was parents’ desire to protect their children and society from infectious diseases. Whereas our study confirmed that vaccine-hesitant parents were mostly concerned about already occurring side effects or their risk. Although the discussion about mandatory vaccination has been trending for a while, there was no consensus about mandatory children vaccination in Lithuania, as the opinions of the respondents were distributed almost evenly. Our findings confirmed that the formation of parents’ views regarding childhood vaccination is a complex process; consequently, sociodemographic and country-related variables should be considered in the national vaccination policy-making process while still maintaining parental autonomy.
